# Uptake of hepatitis C direct-acting antiviral treatment in China: a retrospective study from 2017 to 2021

**DOI:** 10.1186/s40249-023-01081-4

**Published:** 2023-03-28

**Authors:** Xinyu Du, Jiarun Mi, Hanchao Cheng, Yuanyuan Song, Yuchang Li, Jing Sun, Polin Chan, Zhongdan Chen, Simon Luo

**Affiliations:** 1 School of Health Policy and Management, Chinese Academy of Medical Sciences and Peking Union Medical College, Dongdansantiao, Dongcheng District, Beijing, 100730 China; 2 Hepatitis/TB/HIV/STI, World Health Organisation Regional Office for the Western Pacific, P.O. Box 2932, 1000 Manila, The Philippines; 3 Hepatitis/TB/HIV/STI, World Health Organisation Representative Office in China, 401 Dongwai Diplomatic Building 23, Dongzhimenwai Dajie, Chaoyang District, Beijing, 100600 China; 4 IQVIA Holding Company, 138 Wangfujing street, Xindongan Palza, Block 3, Beijing, 100006 China

**Keywords:** Direct-acting antiviral, Hepatitis C, Treatment, Universal health insurance, China

## Abstract

**Background:**

Direct-acting antivirals (DAAs) for hepatitis C treatment in China became available since 2017. This study expects to generate evidence to inform decision-making in a nationwide scale-up of DAA treatment in China.

**Methods:**

We described the number of standard DAA treatment at both national and provincial levels in China from 2017 to 2021 based on the China Hospital Pharmacy Audit (CHPA) data. We performed interrupted time series analysis to estimate the level and trend changes of the monthly number of standard DAA treatment at national level. We also adopted the latent class trajectory model (LCTM) to form clusters of the provincial-level administrative divisions (PLADs) with similar levels and trends of number of treatment, and to explore the potential enablers of the scale-up of DAA treatment at provincial level.

**Results:**

The number of 3-month standard DAA treatment at national level increased from 104 in the last two quarters of 2017 to 49,592 in the year of 2021. The estimated DAA treatment rates in China were 1.9% and 0.7% in 2020 and 2021, which is far below the global target of 80%. The national price negotiation at the end of 2019 resulted in DAA inclusion by the national health insurance in January 2020. In that month, the number of treatment increased 3668 person-times (*P* < 0.05). LCTM fits the best when the number of trajectory class is four. PLADs as Tianjin, Shanghai and Zhejiang that had piloted DAA price negotiations before the national negotiation and that had explored integration of hepatitis service delivery with prevention and control programme of hepatitis C within the existing services demonstrated earlier and faster scale-up of treatment.

**Conclusions:**

Central negotiations to reduce prices of DAAs resulted in inclusion of DAA treatment under the universal health insurance, which are critical elements that support scaling up access to hepatitis C treatment in China. However, the current treatment rates are still far below the global target. Targeting the PLADs lagged behind through raising public awareness, strengthening capacity of the healthcare providers by roving training, and integrate prevention, screening, diagnosis, treatment and follow-up management of hepatitis C into the existing services are needed.

**Supplementary Information:**

The online version contains supplementary material available at 10.1186/s40249-023-01081-4.

## Background

Hepatitis C is one of the major global public health threats. Currently, 0.7% of the world’s population (56.8 million people) are infected with hepatitis C virus (HCV), which remains a leading cause of chronic hepatitis, cirrhosis, liver failure and hepatocellular carcinoma [1], accounting for more than 0.3 million lives lost each year [2]. The introduction of direct-acting antivirals (DAAs) with high cure rates, shorter treatment durations, fewer side-effects as compared to previous medicines, revolutionized the treatment of HCV infection, and provide an unprecedented opportunity for widespread scale-up and elimination [3, 4].

In 2016, the World Health Organization (WHO) set goals for HCV elimination, defined as a 65% reduction in mortality and a 90% reduction in incidence of new infections by 2030 [5, 6]. Reaching these goals will require a large scale-up of HCV testing to diagnose and treat 80% of all the people living with HCV. In 2015, only 7.4% (1.1 million) of the diagnosed HCV-infections started treatment. The global cumulative number of persons treated for HCV was 5.5 million, only about half of them had received DAA treatment, and there were more new HCV infections than patients who had started on treatment. Recent new data show that 9.4 million people diagnosed with HCV infections had been treated using DAAs globally between 2015 and 2019 [7]. Although a number of developing countries have been making substantial progress and move towards elimination of hepatitis C, there has been a dearth of national or provincial level information on HCV care in China [8–11].

China is one of the countries with the largest number of people living with chronic HCV (estimated 7.6 million) in the world [12], only 25% of them are diagnosed and 1% treated [1]. In response to the goal of viral hepatitis elimination as a public health threat by 2030, China implemented a series of national policies to improve the availability and affordability of DAAs and scale-up of DAA treatment at both national and provincial levels. Some provincial-level administrative divisions (PLADs) and cities had already been exploring public funding of DAAs even before the national initiative and changes in local policies including piloting innovative health insurance financing models. Other PLADs initiated pilots where the primary care services provided hepatitis C care through improving the family doctor contract services mechanism, as well as integration of hepatitis C management into the existing disease prevention and control programs (Additional file 1: Annex 1). Several DAAs including locally developed ones were fast-tracked for market entry in 2017. Eight DAAs are currently funded by the national health insurance following central price negotiations since 2019. The cost of DAA treatment in China decreased to USD 200–1500 for a 12-week course of cure, and the individual out-of-pocket expenditure is within USD 150–450 per 12-week course of cure [13].

To understand the number of DAA treatment at either provincial level or the national level, we used the terms of "Hepatitis C” or “HCV” and "treatment" to search PubMed, Embase, and CNKI databases from January 1, 2017, to December 31, 2022 in English and Chinese. We only identified a very limited number of studies reporting the number of treatment of hepatitis C in China either at national or local levels. China started to monitor the prevention and treatment of hepatitis C in sentinel hospital since 2016, which has been expanded to 58 secondary and tertiary hospitals in 13 PLADs [14–16]. At the provincial level, only Tianjin City reported the number of DAA treatment in designated hospitals [17]. There is no information on the number of people living with HCV under or received DAA treatment at national levels due to the absence of a national patient monitoring or registry system. Studies that monitor the treatment of hepatitis C were all in specific hospitals or within limited areas [18–20].

To the best of our knowledge, this is the first comprehensive analysis attempted to estimate the number of DAA treatment at national level, and to explore the potential enablers of the scale-up of DAA treatment at provincial level in China. We evaluated the level and trend changes of monthly number of standard DAA treatment before and after the first batch of DAAs funded by the national health insurance following price negotiation in January 2020. We also compared the quarterly number of 3-month standard DAA treatment across PLADs from quarter 1 (Q1) 2020 to quarter 4 (Q4) 2021, and analysed the enablers of scale-up the DAA treatment at local level. The study expects to generate evidence to inform decision-making in a nationwide scale-up of DAA treatment in China, and contribute to the achievement of the 2030 goal.

## Methods

### Data source

This study extracted longitudinal monthly procurement data of all marketed DAAs in China (Additional file 1: Annex 2) from QuintilesIMS Health (IQVIA)’s China Hospital Pharmacy Audit (CHPA). CHPA collects medicines procurement data from health facilities with more than 100 beds in 31 PLADs of China (including county hospitals in rural areas). IQVIA’s medicines procurement data is an established system and is widely used in research for pharmaceutical policy and market analyses [21–29]. Eleven DAAs and their combinations were identified from CHPA between July 2017 (when the first DAA was approved for market in Chinese mainland) to December 2021. We transformed the procurement data of DAAs into the number of standard DAA treatment of hepatitis C following the pharmaceutical manufacturers' instructions approved by the national regulatory authority [30] and the National Guidelines for Hepatitis C Prevention and Treatment [31]. We obtained the COVID-19 epidemic data and other data from the website of National Health Commission [32], China Statistical Yearbook 2021 [33], China Health Statistical Yearbook 2021 [34] and China Health Account Report 2021 [35].

### Measurement

We calculated the monthly number of standard DAA treatment at national level for the time series statistical analysis. Considered that the course of the standard DAA treatment is 12-week, we also calculated the quarterly number of 3-month standard DAA treatment at both national and provincial levels (not included Tibet) to demonstrate the number of patients completed the 12-week standard treatment.

Provincial level monthly number of standard DAA treatment = ${\sum_{i=1}^{n}}DAA \, combination_{i}$, n is the number of DAA combinations identified from CHPA in specific month.

Provincial level quarterly number of 3-month standard DAA treatment = (∑c January–March)/3 or (∑c April–June)/3 or (∑c July–September)/3 or (∑c October–December)/3.

National level data = $\sum_{i=1}^{n}provincial \, level$, *n* = 30, is the number of PLADs included in this study.

#### General conditions for conversion of monthly DAA volumes to monthly number of standard DAA treatment

If the monthly procurement volumes of individual components of a DAA combination match with each other, the monthly number of standard treatment with that DAA combination was estimated based on the monthly procurement volume of any one of individual components. If the monthly procurement volumes of individual components of a DAA combination do not match with each other, the monthly number of standard treatment with that DAA combination was estimated based on the highest monthly procurement volume of individual component. The gap volumes of the other components were deducted from their consumption volumes in the next month.

#### Specific conditions for conversion of monthly DAA volumes to monthly number of standard DAA treatment

Daclatasvir (DAC), asunaprevir (ASV) and sofosbuvir (SOF) were the first batch of DAAs widely used in China. The monthly number of treatment with DAC + ASV was estimated based on the monthly procurement volume of ASV, and the surplus of DAC was used to estimate the number of standard treatment with SOF/DAC. The surplus of SOF was used to estimate a small number of treatment in case SOF was in combination with pegylated interferon alfa and ribavirin (PR) or ribavirin (RBV). The dosages of SOF in both treatments are the same. Fixed-dose combinations including SOF all have matched volumes of individual components.

The annual reported incidence of hepatitis C and total population in each PLAD describe provincial epidemiological and demographic characteristics. The per capita gross domestic product (GDP/capita), per capita total health expenditure (THE/capita), and the proportionate population covered by the urban employee health insurance in each PLAD reflect provincial socio-economic development status. The proportionate health expenditure of public health institutions represents public inputs to public health institutions (including Centers for Disease Control and Prevention, etc.). And the proportionate patient out-of-pocket (OOP) expenditure reflects individual financial burden of healthcare (The sources of these provincial data were listed in Additional file 1: Annex 3).

### Study design

**Fig. 1:**
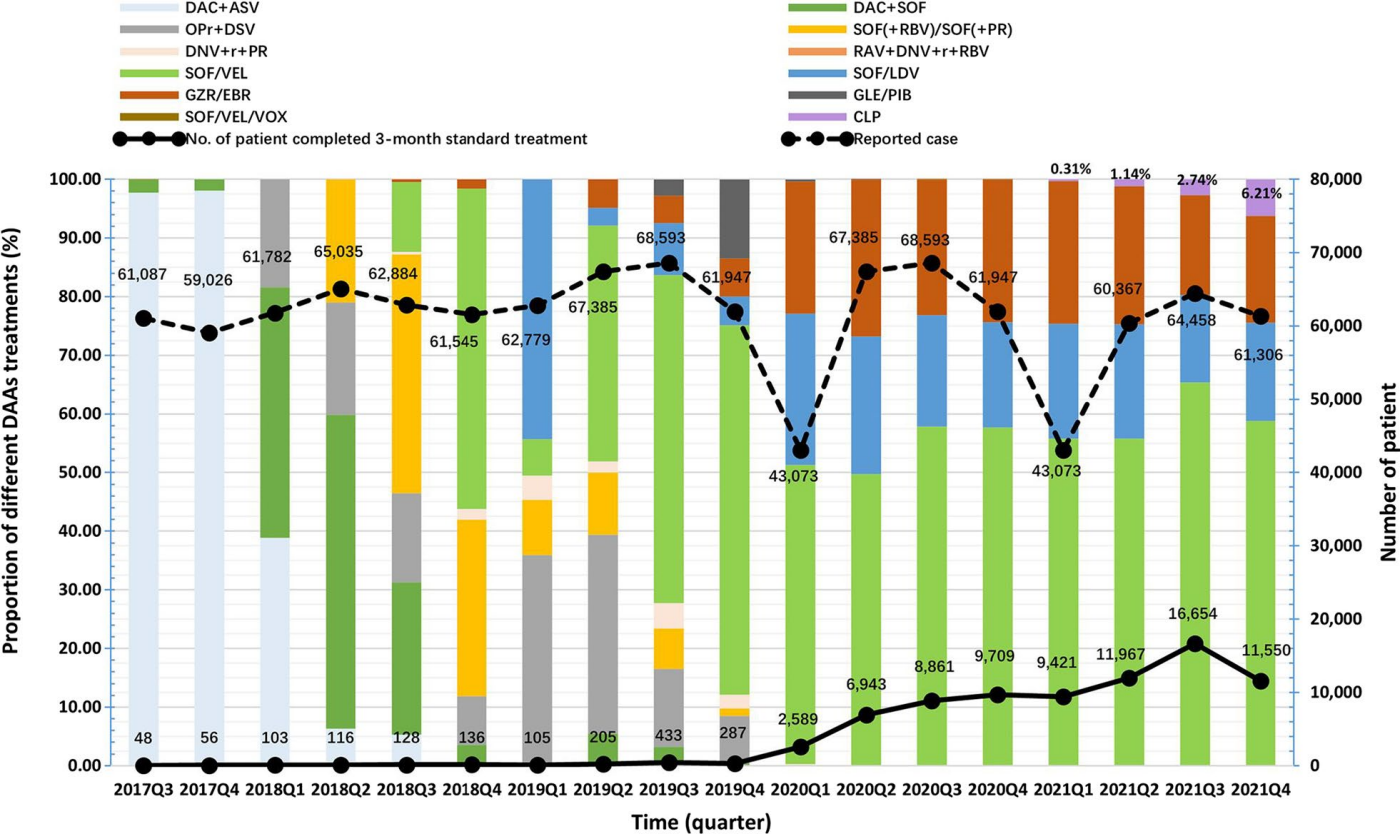
Number of 3-month standard DAAs treatment and reported cases of hepatitis C (Q3 2017–Q4 2021). *ASV* asunaprevir, *CLP* coblopasvir, *DAC* daclatasvir, *DNV* danoprevir, *DSV* dasabuvir, *GLE/PIB* Glecaprevir/Pibrentasvir, *GZR/EBR* Grazoprevir/Elbasvir, *OPr* Ombitasvir/Paritaprevir/Ritonavir, *PR* pegylated interferon alfa/ribavirin, *r* ritonavir, *RAV* ravidasvir, *RBV* ribavirin, *SOF* sofosbuvir, *SOF/LDV* sofosbuvir/ledipasvir, *SOF/VEL* sofosbuvir/velpatasvir, *SOF/VEL/VOX* sofosbuvir/velpatasvir/voxilaprevir

We firstly drew the time series chart of the quarterly number of 3-month standard DAA treatment of hepatitis C at national level from Q3 2017 to Q4 2021, and the cumulative histograms for each of the DAA combinations (Fig. 1). We also drew the time series charts of the monthly number of standard DAA treatment at national level from July 2017 to December 2021 (Additional file 1: Annex 4), as well as the quarterly number of 3-month standard DAA treatment for each of 30 PLADs from Q1 2020 to Q4 2021 (Additional file 1: Annex 5). Based on the national level monthly data from July 2017 to December 2021, we performed interrupted time series (ITS) analysis to estimate the immediate level changes and afterwards trend changes of the monthly number of standard DAA treatment in the month that the first batch of DAAs were funded by the national health insurance in January 2020, and in the month that the first domestically developed DAA was funded by the national health insurance in March 2021, respectively. Based on the quarterly panel data of number of 3-month standard DAA treatment of hepatitis C of 30 PLADs from Q1 2020 to Q4 2021, we adopted Latent Class Trajectory Model (LCTM), a specialised form of finite mixture modelling to simplify heterogeneous PLADs into more homogeneous clusters, and identify latent classes of PLADs following similar progressions of determinants over time [36]. We listed the PLADs in each of the identified trajectories class, and their respective characteristics (epidemiological and demographic, socioeconomic development, public input in public health institutions, and the individual financial burden of healthcare) in a heat map, in order to identify any common characteristics within the class and differences across classes. We also linked the pilot explorations of some areas with the clustering results, and analysed the potential factors that might affect the scale-up of DAA treatment.

### Statistical analysis

We fitted the following segmented liner regression model with the monthly number of standard DAA treatment at national level from July 2017 to December 2021. The two policy intervention time points were January 2020 when the first batch of DAAs funded by the national health insurance, and March 2021 when the first locally developed DAA was funded by the national health insurance.

$${Y}_{t} = {\beta }_{0} + {\beta }_{1}{X}_{1} + {\beta }_{2}{X}_{2} + {\beta }_{3}{X}_{3} + {\beta }_{4}{X}_{4} +{ \beta }_{5}{X}_{5} + {\beta }_{6}{X}_{6} + {\varepsilon }_{t},$$

*X*_*1*_ is a time variable, denotes the number of months from 1 to 54 (from July 2017 to December 2021). *X*_*2*_ and *X*_*4*_ are policy variables. Before January 2020, *X*_*2*_ = 0; after January 2020, *X*_*2*_ = 1. Between January 2020 and March 2021 *X*_*4*_ = 0; after March 2021, *X*_*4*_ = 1. *X*_*3*_ and *X*_*5*_ are time variables, denote the number of months after January 2020 and after March 2021 respectively. *Yt* denotes the monthly number of standard DAA treatment. Considering the possible impact of coronavirus disease 2019 (COVID-19) pandemic, we included the national monthly number of new confirmed COVID-19 cases in the model as a control variable ${X}_{6}$. ${\beta }_{1} \, \text{denotes the baseline trend}. {\beta }_{2}$ and ${\beta }_{4}$ denote the immediate level changes of the monthly number of standard DAA treatment in January 2020 and March 2021, respectively. ${\beta }_{3}$ and ${\beta }_{5}$ denote the trend changes of the monthly number of standard DAA treatment between January 2020 and March 2021, and after March 2021, respectively.

To identify the PLADs with similar trends of the quarterly number of 3-month standard DAA treatment, we adopted LCTM to identify the potential trajectory categories of the quarterly number of 3-month standard DAA treatment of 30 PLADs from Q1 2020 to Q4 2021. We added the quadratic term of "quarter" in the model, in order to fit the model for non-liner trajectories better. We calculated the posterior probability of the trajectory of each PLAD. The model with the lowest Akaike information criterion (AIC) value was selected as the best fitted one. Meanwhile, we secured that the posterior probability should be greater than 0.7, and the number of PLADs in each trajectory class to total number of PLADs (30) should be greater than 2% [37].

For all analyses, a two-tailed *P* value < 0 0.05 was considered statistically significant. We performed all statistical analyses with software R 4.2.0 (Lucent Technologies, Jasmine Mountain, USA) and Microsoft Excel 2019.

## Results

### Number of DAA treatment at national level

The number of reported cases captured by the National Infectious Diseases Reporting System has been always keeping at around 60,000 per quarter, except the first quarters of 2020 and 2021 during the COVID-2019 pandemic. From July 2017 to December 2021, the total number of 3-month standard DAA treatment in China was 79,321, including 28,102 in 2020 and 49,592 in 2021. Before 2020, the monthly number of DAA treatment was noted to be low. However, since January 2020, the numbers of treatment accelerated rapidly. Three combinations of DAA regiments including sofosbuvir/ledipasvir (SOF/LDV), sofosbuvir/velpatasvir (SOF/VEL) and elbasvir/grazoprevir (GZR/EBR) acounted of almost all (nearly 100%) of the HCV treatment since January 2020 when the regiments began to be publicly funded (that is, included under the national health insurance coverage). Treatment numbers of the pan-genotype combination (SOF/VEL) is consistently the highest. By Q4 2021, the market share of the domestically developed coblopasvir (CLP) was no more than 7%, even though it was publicly funded since March 2021 (Fig. 1).

### Level and trend changes of the number of monthly DAA treatment at national level

The ITS analysis results are shown in Table 1 and Additional file 1: Annex 4 where from January 2020 when the first batch of DAA regiments were included under the national health insurance. This resulted in a 3668 person-times increase in standard DAA treatment compared to before (${\beta }_{2}$, *P* < 0.05). Thereafter, between January 2020 and March 2021, numbers of treatment increased at 551 person-times/month (${\beta }_{3}$, *P* < 0.001). We noted that the market entry of the first domestically developed CLP which was included under national health insurance did not substantially change the numbers of treatment in March 2021 (${\beta }_{4}$, ${ \beta }_{5}$, *P* > 0.05), when it was introduced.

**Table 1:** ITS regression results of the monthly number of standard DAA treatment at national level (July 2017–December 2021)

	Estimate	Std. error	*t*-value	*P*-value
${\beta }_{0}$	− 6.08	743.66	− 0.008	0.99
${\beta }_{1}$	10.83	41.89	0.26	0.80
${\beta }_{2}$	**3667.72**	1411.20	2.60	**0.01**
${\beta }_{3}$	**550.94**	153.32	3.59	** < 0.001**
${\beta }_{4}$	− 526.13	1642.81	− 0.32	0.75
${\beta }_{5}$	− 186.72	266.08	− 0.70	0.49
${\beta }_{6}$	− 0.04	0.03	− 1.21	0.23

The value of Durbin-Watson statistic is 1.92

Values in bold are significant (*P*<0.05)

ITS: interrupted time series

### Trajectory categories of the quarterly number of 3-month standard DAA treatment at provincial level

**Fig. 2:**
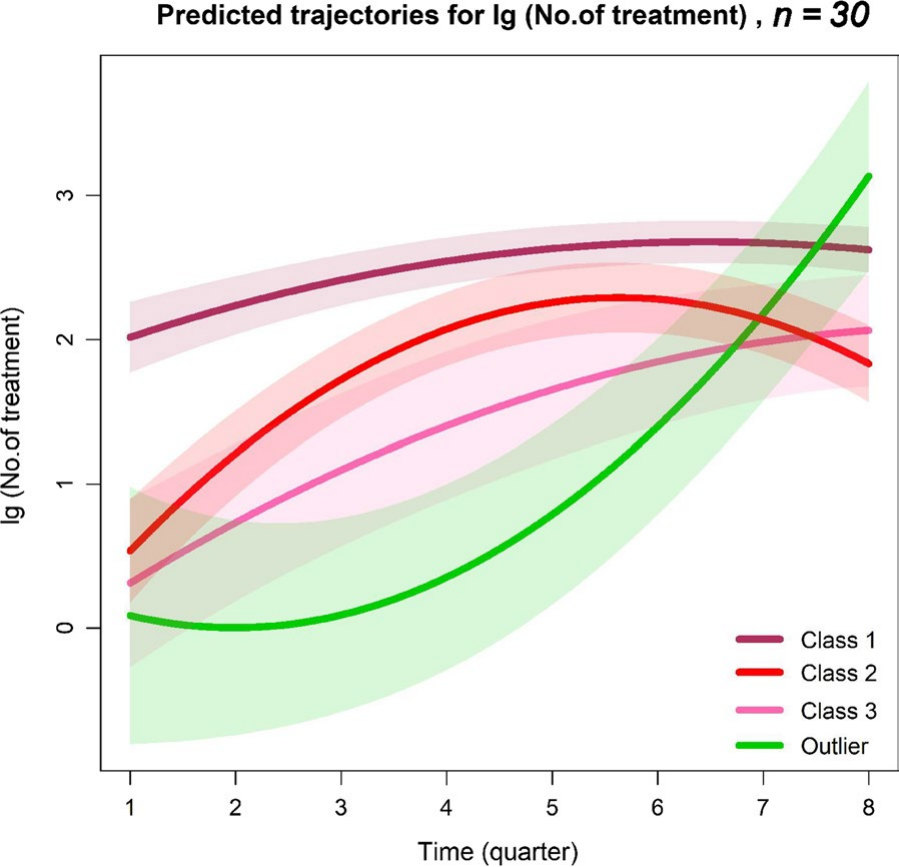
Trajectory class-specific mean predicted trajectory

**Fig. 3:**
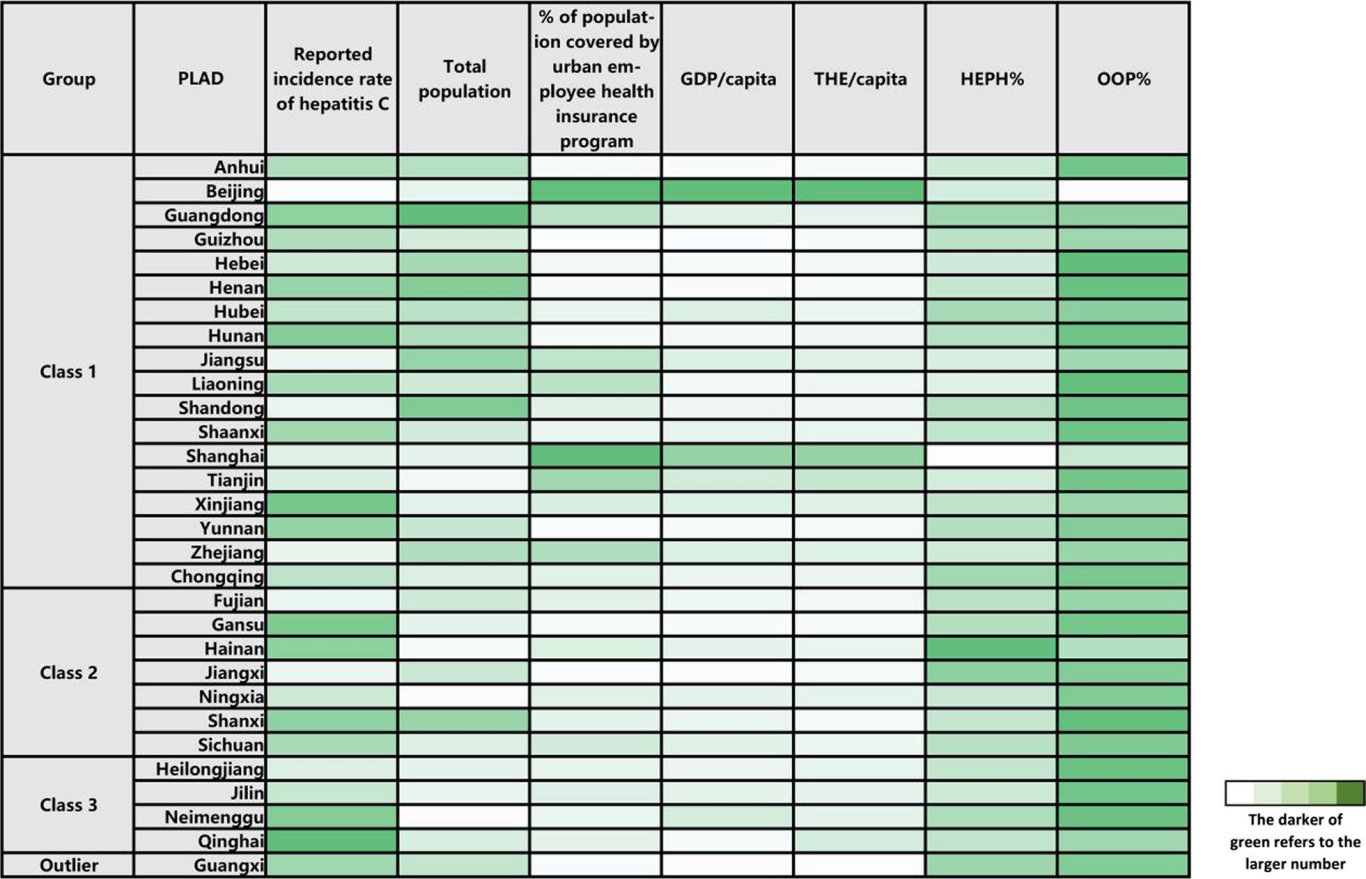
Heat map of demographic, socioeconomic and epidemic characteristics of 30 PLADs (2020). The darker the color, the larger the number; *PLAD* provincial-level administrative division, *GDP* gross domestic product, *THE* total health expenditure, *HEPH%* proportionate health expenditure of public health institutions, *OOP%* proportionate out-of-pocket expenditure

As presented in Additional file 1: Annex 6, LCTM fits the best when the number of trajectory class is four. We defined the four trajectory classes as class 1, class 2 and class 3, and an outlier. The predicted trajectories of the four classes were presented in Fig. 2. Class 1 includes the PLADs of Anhui, Beijing, Guangdong, Guizhou, Hebei, Henan, Hubei, Hunan, Jiangsu, Liaoning, Shandong, Shaanxi, Shanghai, Tianjin, Xinjiang, Yunnan, Zhejiang, and Chongqing (in dark-red color). Class 2 includes the PLADs of Fujian, Gansu, Hainan, Jiangxi, Ningxia, Shanxi, and Sichuan (in red color). Class 3 includes the PLADs of Heilongjiang, Qinghai, Jilin, and Inner Mongolia (in light-red color). And the outlier is Guangxi (in green color) PLAD. As showed in Additional file 1: Annex 5, the number of treatment before 2020 in the PLADs of class 1, to class 2, to class 3 demonstrate from earlier growth, to later growth, and very late growth, respectively. The outlier PLAD (Guangxi) was always near to 0 before January 2021, and kept at a very low level until Q2 2021, when the other PLADs already grew to a high level. The growth of the outlier PLAD emerged in Q3 2021. As presented in Fig. 3, the four PLADs in class 3 and the outlier PLAD have the similar characteristics of lower economic development level but higher hepatitis C incidence rate (as documented in the National Notifiable Infectious Disease Reporting System).

## Discussion

2017 is the first year that the first DAA combination (DAC tablet and ASV capsule) were marketed for treatment of adult chronic hepatitis C in Chinese mainland [38]. DAA options gradually increased afterwards. China updated the national guideline for the prevention and treatment of hepatitis C in 2019, which recommended interferon-free pan-genotypic DAAs. However, scale-up of DAA treatment was not seen until January 2020, when the first batch of DAA combinations were publicly funded (included under national health insurance coverage) following national price negotiations, which reduced the prices by over 85%. After January 2020, the number of DAA treatment began to increase significantly at national level and in the PLADs in the high-level treatment trajectory class. This implied a significant positive impact of the affordability of DAA on the scale-up of DAA treatment.

Domestically developed DAAs started to be marketed in Chinese mainland since 2018. Among them, CLP was immediately funded by the national health insurance in March 2021. However, public funding of CLP did not have significant impact on the scale-up of DAA treatment. The reason might be that the local developer of CLP was not able to market it as a pan-genotype combination with SOF, the corner-stone of pan-genotype DAA combinations. Although the local producer of CLP already registered generic SOF with the Chinese drug regulatory authority, they are still not able to formally market it. This is because of patent protection of the originator until 2024. Currently, none of the domestically developed DAAs can be marketed as a pan-genotype combination with SOF, and the national health insurance is limited in the indication of domestically developed DAAs to specific genotype instead of pan-genotype. Genotyping of the HCV virus is not publicly funded in most areas of China. Compared with imported pan-genotype combinations, the price advantage, as well as the overall market competitiveness of domestically developed DAAs may be lesser. Compared with the global lowest price of the generic DAAs in other middle-income countries like Egypt and Malaysia, where the 12-week treatment cost has been below USD 40 [13], the current prices of DAAs in China are still too high. For the patients with low ability-to-pay, especially those entitled to lower health insurance benefits, may still have specific economic problems and therefore have reduced willingness for treatment [39]. Marketing the domestically developed pan-genotype DAA combinations immediately after the patent expiring of sofosbuvir could be a good bargaining leverage to further reduce the prices of originated DAAs. In addition, improving the safety net for those who have poor ability-to-pay will contribute to universal access to DAA treatment.

Previous studies estimated that there are about 4–5 million chronic HCV infections who need treatment in China [15]. Based on the number of 3-month standard DAA treatment estimated from this study, the DAA treatment rates in China was 1.9% and 0.7% in 2020 and 2021 respectively, which were approximate the 1% treatment rate estimated by previous studies [1]. These numbers are far below the 2030 targets (80% eligible received treatment) [40]. The highest number of 3-month standard DAA treatment is only one fourth of the reported national notifiable HCV cases during the same quarter. Compared to low- and middle-income countries such as Georgia and Egypt, where the treatment rates of active hepatitis C infections have reached a high level (79% and 92% of the people with active HCV infection initiated treatment in 2018–2019, respectively) [9, 10], there is still a long way for China to go.

Affordability is important for further scale up DAA treatment, but it is not the only issue. Countries such as Australia which provide universal health coverage including HCV treatment have shown that while subsidized treatment is necessary, it is insufficient to reach the elimination targets [41]. Although new hepatitis C prevention and treatment guideline recommend DAA as the first-line treatment option for hepatitis C in 2019 [31], we noted a number of PLADs with lower economic development level and high burden of hepatitis C in class 3 and the outlier PLAD showing low levels of treatment uptake. This might be associated with the lack of knowledge and awareness of hepatitis C in the less developed areas among the public service providers [42]. As well, insufficient availability of DAAs in hospitals in these areas due to delayed implementation of the national policy may result in delayed scale-up of treatment [43]. For the PLADs lagged behind, raising public awareness, strengthening capacity of the healthcare providers through roving training by the trainers of new guidelines from the advanced area are urgently needed.

PLADs in class 1 demonstrated earlier increase in the number of DAA treatment even before 2020. This is mainly because of the pilots of local price negotiations and local insurance funding of DAAs before the national initiative. These PLADs piloted multiple projects to explore pathways to integrate hepatitis C management program into the existing services. Tianjin, for example, started to have all the national guideline recommended DAAs funded by local health insurance in 2018, and implemented an innovative capitated payment for outpatient care of hepatitis C [44]. The family doctor contract gatekeeping system in Shanghai was improved through the health system reform since the early 2010s, and Shanghai was the first area to incorporate hepatitis C management into the scope of family doctor contract services. Primary care centers provide free physical examination, chronic disease monitoring, prescription filling, health consultation, and infection prevention guidance. Shanghai also integrated hepatitis C with the prevention and control program of HIV/AIDS, which provides voluntary counseling and testing, free rapid screening, health consultation, referral to specialized hospitals for diagnostic test and treatment, and follow-up visits. Some districts of Shanghai provide free RNA test for the HCV-antibody positives detected from the free rapid screen [45, 46]. Ningbo City of Zhejiang Province built a ‘four-in-one’ initiative for hepatitis C management in 2019, which enhanced the role of primary care institution, and its cooperation with the Center for Disease Prevention and Control, insurance designated hospitals, and the health administrative department. Ningbo established a feasible referral and follow-up mechanism based on the functioning primary care services to form the closed-loop management of hepatitis C [47]. These local pilots before 2020 in the ‘pioneering’ PLADs in the high-level treatment trajectory class led them being at the forefront of rapidly scaling up the DAA treatment, when the policy environment changed. In the other PLADs within the same trajectory class, although the number of DAA treatment was relatively low before 2020, these PLADs responded quickly when the DAAs started to be publicly funded.

WHO highlighted the need to bring hepatitis care closer to primary care and communities so that people have better access to treatment and care [48]. The pilots in Ningbo of Zhejiang and Shanghai focusing on supporting comprehensive primary care response for HCV treatment well follow the above direction pointed out by WHO, and should be expanded to other areas of China. This direction has been practiced in many countries that have substantially increased DAA treatment access by building on existing community-based services and promoting simplified service delivery models. Decentralizing testing and treatment to lower levels of care, integrating with other services, and task-sharing with delivery of care and treatment by non-specialists and nurses are among the key strategies [49] and have been implemented successfully in low- and middle-income countries such as Egypt, Georgia, Cambodia and Malaysia. In 2019, Egypt implemented their universal hepatitis C program that improved awareness among citizens, provided free and accessible HCV testing, infection control, treatment and follow-up. The free screening program integrated non-communicable disease and viral hepatitis, and included tests for diabetes, obesity and blood pressure, as well as hepatitis B vaccination. The HCV testing covered 62 million adults and 15 million adolescents, and 92% of the 1.15 million HCV seropositive people who have completed testing have received DAA treatment [9]. Georgia conducted free HCV antibody testing for blood donors and HIV-infections, and integrated screening, care and treatment in the primary care settings and harm-reduction centers throughout the country. The integration allowed patients and harm reduction beneficiaries to receive hepatitis C care and treatment services in familiar and convenient locations [10]. Medicines Sans Frontieres established a simplified management model of hepatitis C based on the community level health facilities in Cambodia since 2016. The integrated model has transferred many clinical tasks of hepatitis C from doctors to nurses and pharmacists, which provided testing for about 135,000 people and treatment for 18,000 people by the end of 2020 [50]. As a middle-income country, Malaysia integrated its viral hepatitis programme into the national HIV programme since 2017 [51]. Simplifying the treatment process, integrating hepatitis C prevention and treatment so that general practitioners at the primary care clinics can test, confirm and treat, have supported the country’s progress towards elimination by 2030. Spain provided patients with hepatitis C in primacy care together with the one-step diagnosis to effectively detect hidden infections and increased the number of treatments [52]. South-West England integrated an algorithm into primary care information technology systems to identify individuals with high-risk markers of HCV, and rolled out this alongside educational and training packages for staff. The system is expected to be scale up across the UK [53]. Studies in the United States proved that as patients were typically more engaged with their primary care provider, hepatitis C primary care treatment program were in a good position to identify and treat hepatitis C, and especially an effective way to treat HCV infection in underserved communities [54–59]. These successful experiences of integration of prevention, screening, diagnosis, treatment and follow-up management of hepatitis C into the existing services provide important lessons for China to further scale up DAA treatment.

The findings of this study are novel and meaningful for decision-makers to recalibrate the public policy towards elimination of hepatitis C. While the study has several limitations. First, the hospital medicines procurement data may not accurately reflect the actual clinical use due to that the remaining medicines purchased in the current month may be used in the next month, which may lead to underestimation of the actual consumption and overestimation of the previous month. Adopting the quarterly data could address this problem to some extent. Second, CHPA only collects data from hospitals with more than 100 beds, and medicines used in primary care and retail pharmacies are not included in the statistics. Considering that the diagnosis and treatment of hepatitis C in China are mainly carried out in specialized or general tertiary hospitals, CHPA data at this time can reflect the overall consumption of DAAs. Third, this study assumes that all patients followed the 3-month consecutives standard DAA treatment as recommended by the national guideline, which does not consider non-standard therapy in the real-world setting. Fourth, although ITS regression analysis results show that the impact of the COVID-19 outbreak on the number of monthly DAA treatments was not statistically significant, further in-depth study is needed. Last, the analysis was based on the number of treatment measurements of 30 PLADs over eight quarters. Limited numbers of observations may also restrict the statistical power of LCTM analysis. If patient information is accessible, comprehensive in-depth regression analysis of the factors that affect the DAA treatment of hepatitis C could be performed.

## Conclusions

Central negotiations to reduce prices of DAAs resulted in inclusion of DAA treatment under the universal health insurance, which are critical elements that support scaling up access to hepatitis C treatment in China. However, current treatment rates are still far below the global target. Targeting the PLADs lagged behind through raising public awareness, strengthening capacity of the healthcare providers by roving training, and integrate prevention, screening, diagnosis, treatment and follow-up management of hepatitis C into the existing services are needed.

## Supplementary Information

**Additional file 1.** Supplementary tables and figures.

## Data Availability

The data that support the findings of this study are available from IQVIA but restrictions apply to the availability of these data, which were used under license for the current study. Data will be however available upon request from the corresponding author to get permission of IQVIA with appropriate non-commercial use reasons.

## Abbreviations

DAA: Direct-acting antiviral
LCTM: Latent class trajectory model
HCV: Hepatitis C virus
WHO: World Health Organization
CHPA: China Hospital Pharmacy Audit
GDP: Gross domestic product
THE: Total health expenditure
OOP%: Proportionate out-of-pocket
HEPH%: Proportionate health expenditure of public health institutions
ITS: Interrupted time series
AIC: Akaike information criterion
DAC: Daclatasvir
ASV: Asunaprevir
SOF: Sofosbuvir
OPr: Ombitasvir/paritaprevir/ritonavir
r: Ritonavir
DSV: Dasabuvir
RBV: Ribavirin
PR: Pegylated interferon alfa/ribavirin
DNV: Danoprevir
RAV: Ravidasvir
VEL: Velpatasvir
LDV: Ledipasvir
GZR/EBR: Grazoprevir/elbasvir
GLE/PIB: Glecaprevir/pibrentasvir
VOX: Voxilaprevir
CLP: Coblopasvir
HIV: Human immune-deficiency virus
AIDS: Acquired immune deficiency syndrome
UK: United Kingdom
