# Supplementary material for: Uptake of hepatitis C direct-acting antiviral treatment in China: a retrospective study from 2017 to 2021
Source: Infect Dis Poverty. 2023 Mar 28;12:28. doi: 10.1186/s40249-023-01081-4 (PMC10043849; doi:10.1186/s40249-023-01081-4)
Supplement: Supplementary file 1 — Additional file 1. Supplementary tables and figures. [file 40249_2023_1081_MOESM1_ESM.docx]

**Uptake of Hepatitis C direct-acting antiviral treatment in China：a retrospective study from 2017 to 2021**

**March:** 3 imported novel DAAs (EBR/GZR, SOF/LEV, SOF/VEL) and 1 local novel DAA CLP) covered by the national health insurance through price negotiation ^[15]^

**May:** Dispense of insurance covered DAAs by designated retail pharmacies encouraged; ^[16]^

**Sept:** Action Plan of Elimination of HCV as a public health threat (2021–2030);^[17]^

**Apr:** Outpatient DAA treatment of HCV included in the disease-based provider payment in Zhejiang province;^[9]^

**Dec:** Prevention & treatment guideline (2019)^[10]^

Public Health Action Plan to Eliminate Hepatitis C in Ningbo^[11]^

**Apr:** 1^st^ DAA (DAC+ASV) approved for treatment of HCV;^[1]^

**Nov:** Prevention & treatment plan of viral hepatitis (2017–2020)^[2]^

**2022**

**2021**

**2020**

**2019**

**2018**

**2017**

**Jan:** 2 imported novel DAAs (EBR/GZR, SOF/VEL) and 4 local novel DAAs (coblopasvir, danoreivir, ravidavir, emitavir) covered by national health insurance^[18]^

**Jan:** The 1^st^ group of imported novel DAAs (EBR/GZR, SOF/LEV, SOF/VEL) covered by the national health insurance through price negotiation; ^[12]^

Expansion of DAA treatment of HCV in Tianjin;^[13]^

**May：**Shanghai Family Doctor Contract Service Specifications (2020 Edition)；^[14]^

**Jan:** DAAs covered by the public health insurance in Zhejiang province through price negotiation; ^[3]^

**Mar:** Diagnosis standard of HCV;^[4]^

**Apr:** Capitated provider payment of DAA treatment of HCV in Tianjin;^[5]^

**Oct:** SOF/VEL listed by the National EML;^[6]^

**Nov:** DAAs covered by the public health insurance in Chengdu;^[7]^

**Dec**: DAA treatment of HCV included in the low patient co-pay program in Changchun^[8]^

Annex 1 Policies associated with promotion of DAA treatment for hepatitis C (2017–2021)

**Notes:** ASV=asunaprevir; CLP= coblopasvir; HCV=hepatitis C virus; DAA=direct-acting antiviral; DAC=daclatasvir; EBR/GZR=Elbasvir/Grazoprevir; EML=Essential Medicines List; SOF/LDV=sofosbuvir/ledipasvir. Accessed 27 Feb 2023.

1. NMPA. 1^st^ DAA (DAC+ASV) approved for treatment of HCV. <https://www.nmpa.gov.cn/directory/web/nmpa/yaopin/ypjgdt/20170428101301734.html>. Accessed 27 Feb 2023.
2. NHC and other ministries. Prevention & treatment of viral hepatitis plan（2017-2020）. <http://www.nhc.gov.cn/jkj/s3581/201711/aea94a8c1d9d4110a13e2b4d8418c173.shtml>. Accessed 27 Feb 2023.
3. Zhejiang HRSS Administration. DAAs covered by the public health insurance in Zhejiang province through price negotiation. <http://ybj.zj.gov.cn/art/2019/12/12/art_1229113757_564034.html>. Accessed 27 Feb 2023.
4. NHC. Diagnosis Guideline of HCV. <http://www.nhc.gov.cn/wjw/s9491/201803/29997c16d2f24e639ab6c6f55105a9d0.shtml>. Accessed 27 Feb 2023.
5. Tianjin Healthcare and Social Security Administration. Capitated provider payment of DAA treatment of HCV in Tianjin. <http://hrss.tj.gov.cn/zhengwugongkai/zhengcezhinan/zxwjnew/202012/t20201206_4492409.html>. Accessed 27 Feb 2023.

[6] NHC. Release of the 2018 National EML. <http://www.nhc.gov.cn/wjw/jbywml/201810/600865149f4740eb8ebe729c426fb5d7.shtml>. Accessed 27 Feb 2023.[7] Chengdu HR and Social Security Administration. DAAs covered by the public health insurance in Chengdu. http://gk.chengdu.gov.cn/govInfoPub/detail.action?id=102364&tn=6. Accessed 27 Feb 2023.

[8] Changchun HRSS. DAA treatment of HCV included in the low patient co-pay program in Changchun. http://ccrs.changchun.gov.cn/ywdt/zwdt/bjbm/201901/t20190109_1663339.html. Accessed 27 Feb 2023.

[9] Zhejiang HSA. Outpatient DAA treatment of HCV included in the disease based provider payment in Zhejiang province. <http://ybj.zj.gov.cn/art/2019/4/12/art_1615796_33293551.html.> Accessed 27 Feb 2023.

[10] Chinese Society of Hepatology, Chinese Medical Association, Chinese Society of Infectious Diseases, Chinese Medical Association. Guidelines for the prevention and treatment of hepatitis C (2019). J Clin Hepatol. 2019;35(12): 2670-2686.

[11] HC Ningbo. Public Health Action Plan to Eliminate Hepatitis C launched in Ningbo. <http://www.ningbo.gov.cn/art/2019/12/11/art_1229096033_52669279.html>. Accessed 27 Feb 2023.

[12] NHSA & MoHRSS. 1^st^ group of imported novel DAAs (EBR/GZR, SOF/LEV, SOF/VEL) covered by the national health insurance through price negotiation. <http://www.nhsa.gov.cn/art/2019/11/28/art_37_2050.html>. Accessed 27 Feb 2023.

[13] Tianjin HSA. Expansion of DAA treatment of HCV in Tianjin. <http://ylbz.tj.gov.cn/xxgk/zcfg/ybjwj/202010/t20201022_3993988.html.> Accessed 27 Feb 2023.

[14] Shanghai MHC. About the Issuance of the "Shanghai Family Doctor Contract Service Specifications (2020 Edition). <https://wsjkw.sh.gov.cn/jcws2/20200529/a8a87c1294d9413a8b41754056bd59aa.html>. Accessed 27 Feb 2023.

[15] NHSA & MoHRSS.3 imported novel DAAs and 1 local novel DAA covered by the national health insurance through price negotiation.http://www.mohrss.gov.cn/xxgk2020/fdzdgknr/shbx_4216/gsbx/202101/t20210112_407492.html. Accessed 27 Feb 2023.

[16] NHSA & NHC. Dispense of insurance covered DAAs by designated retail pharmacies encouraged. <http://www.nhsa.gov.cn/art/2021/5/10/art_37_5023.html>. Accessed 27 Feb 2023.

[17] NHC, etc. Action plan of elimination of HCV as a public health threat (2021-2030). <http://wjw.lf.gov.cn/zcfg/2535.jhtml>. Accessed 27 Feb 2023.

[18] NHSA & MoHRSS. 2 imported novel DAAs (EBR/GZR, SOF/VEL) and 4 local novel DAAs (CLV, DNV, RAV, EMV) covered by national health insurance. <http://www.nhsa.gov.cn/art/2021/12/3/art_37_7429.html>. Accessed 27 Feb 2023.

Annex 2 All registered DAAs for hepatitis C cure marketed in China by the end of 2021

| INN | Originator company | Market time | Dosage form | Strength & dosage/day | Indication | National health insurance covered indication | EML | RL |
| --- | --- | --- | --- | --- | --- | --- | --- | --- |
| ^a^daclatasvir, DAC | BMS | 2017.04 | Tab | 60 mg | Combined with other DAAs for HCV; not for single use | / | N | N |
| ^a^asunaprevir, ASV | BMS | 2017.04 | Cap | 100 mg × 2 | Combined with daclatasvir for genotype 1b | / | N | N |
| ^a^elbasvir/grazoprevir, EBR/GZR | MSD | 2018.04 | FDC | 50 mg elbasvir + 100 mg grazoprevir | Genotype 1, 2, 3, 4, 5, 6 | Genotype 1, 2, 3, 4, 5, 6 | N | January 2020 |
| ^a^dasabuvir, DSV | Abbvie | 2017.09 | Tab | 250 mg ×2 | Combined with other DAAs for HCV | / | N | N |
| ^a^ombitasvir/paritaprevir/ ritonavir, OPr | Abbvie | 2017.09 | FDC | (12.5 mg ombitasvir + 75 mg paritaprevir +  50 mg ritonavir) ×2 | Combined with other DAAs for HCV | / | N | N |
| ^a^danoprevir, DNV | Asclict | 2018.06 | Tab | 100 mg × 2 | Combined with ravidasvir for genotype 1b | Combined with ravidasvir for genotype 1b | N | January 2022 |
| ^a^ravidasvir, RAV* | Asclict | 2020.07 | Tab | 200 mg | Combined with lopinavir strengthened danoprevir & ritonavir for genotype 1b; not for single use | Combined with lopinavir strengthened danoprevir & ritonavir for genotype 1b; not for single use | N | January 2022 |
| ^a^Sofosbuvir, SOF | Gilead | 2017.09 | Tab | 400 mg | Combined with other DAAs | / | N | N |
| ^a^sofosbuvir/ ledipasvir, SOF/LDV | Gilead | 2018.11 | FDC | 90 mg ledipasvir + 400 mg sofosbuvir | Adult and 12–< 18 | Adult and 12–< 18 | N | January 2020 |
| ^a^sofosbuvir/ velpatasvir, SOF/VEL | Gilead | 2018.05 | FDC | 400 mg sofosbuvir + 100 mg velpatasvir | Genotype 1, 2, 3, 4, 5, 6 | Genotype 1, 2, 3, 4, 5, 6 | Oct 2018 | January 2020 |
| ^a^sofosbuvir/ velpatasvir/  voxilaprevir, SOF/VEL/VOX | Gilead | 2019.12 | Tab | 400 mg sofosbuvir + 100 mg velpatasvir +  100 mg voxilaprevir | Genotype 1, 2, 3, 4, 5, 6 | Genotype 1, 2, 3, 4, 5, 6 | N | January 2022 |
| ^a^glecaprevir/ pibrentasvir, GLE/PIB | Abbvie | 2019.05 | Tab | (glecaprevir 100 mg + pibrentasvir 40 mg) × 3 | Genotype 1, 2， 3, 4, 5, 6 | / | N | N |
| ^a^coblopasvir, CLP | Kwine | 2020.02 | Cap | 60 mg | Combined with SOF for genotype 1, 2, 3, 6 | Not for genotype 1b | N | March 2021 |
| emitasvir, EMV | Changjiang | 2020.12 | Cap | 100 mg | Combined with SOF for Genotype 1 | Combined with SOF for Genotype 1; not for single use | N | January 2022 |

**Notes:** DAAs marked with ^a^ were found in the IQVIA CHPA database; ASV=asunaprevir; CHPA=China Hospital Pharmacy Audit; CLP=coblopasvir; DAC=daclatasvir; DNV=danoprevir; DSV=dasabuvir; EML=essential medicines list; EMV=emitasivr; GLE/PIB=glecaprevir/pibrentasvir; GZR/EBR=grazoprevir/elbasvir; INN= International Nonproprietary Name; IQVIA=QuintilesIMS Health; OPr=ombitasvir/paritaprevir/Ritonavir; PR=pegylated interferon alfa/ribavirin; r=ritonavir; RAV=ravidasvir; RBV=ribavirin; RL= reimbursement list; SOF=sofosbuvir; SOF/LDV=sofosbuvir/ledipasvir; SOF/VEL=sofosbuvir/velpatasvir; SOF/VEL/VOX=sofosbuvir/velpatasvir/voxilaprevir

Annex 3 Sources of provincial epidemiologic, demographic, socio-economic and number of treatment data

| **Variable** | **Data source** |
| --- | --- |
| Reported incidence of hepatitis C | China Health Statistical Yearbook 2021^[1]^ |
| Total population | China Health Statistical Yearbook 2021^[1]^ |
| Proportionate population covered by the urban employee health insurance program | China Health Statistical Yearbook 2021^[1]^ |
| Per capita total health expenditure | China Health Account Report 2021^[2]^ |
| Per capita gross domestic product | China Statistical Yearbook 2021^[3]^ |
| Proportionate health expenditure of public health institutions | China Health Account Report 2021^[2]^ |
| Proportionate patient out-of-pocket expenditure: | China Health Account Report 2021^[2]^ |
| Monthly number of standard DAA treatment | IQVIA, CHPA database |

^[1]^ China National Health Development Research Center. China National Health Accounts Report 2021. Beijing: CNHDRC; 2021.

^[2]^ National Bureau of Statistics of China. China Statistical Yearbook 2021. http://www.stats.gov.cn/tjsj/ndsj/2021/indexch.htm. Accessed 27 Feb 2023.

^[3]^ Yearbook China. China Health Statistics Yearbook 2021. <https://www.yearbookchina.com/navibooklist-n3022013080-1.html.> Accessed 27 Feb 2023.


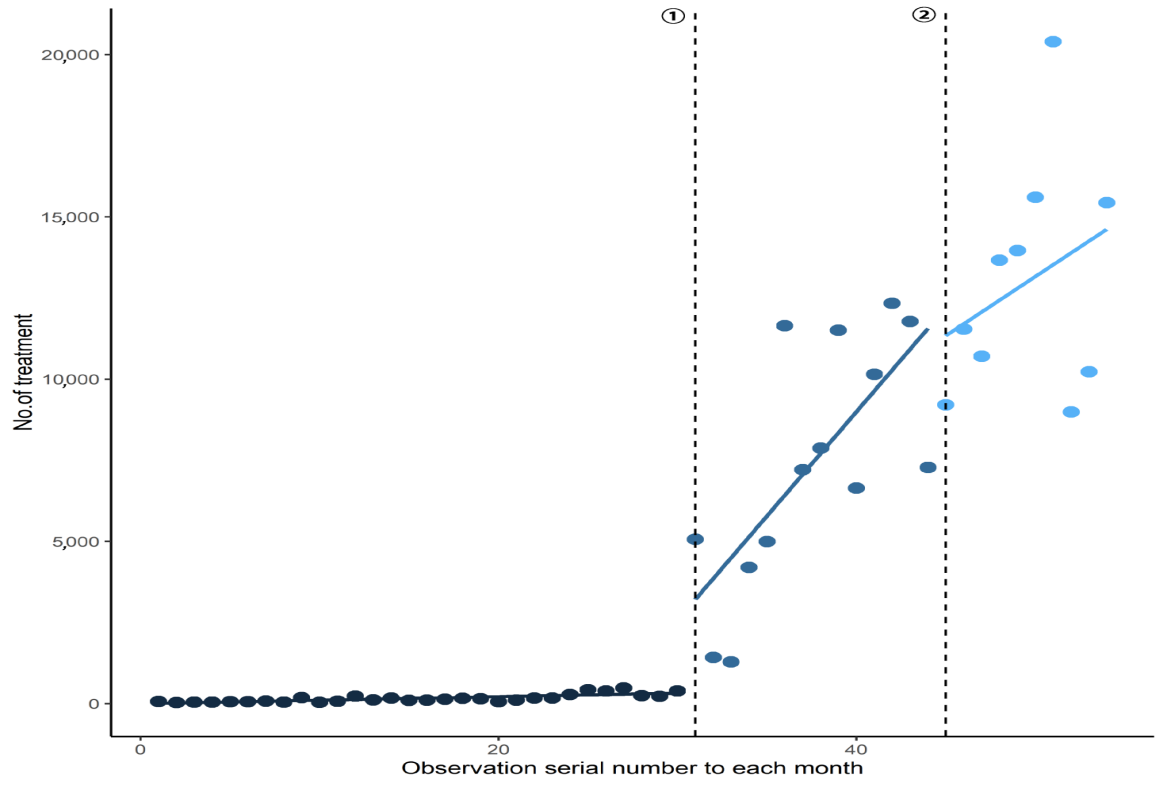


Annex 4 ITS regression results of No. of monthly standard DAA treatment for HCV at national level

**Notes:** ① indicates the first policy intervention time point (January 2020)；

② indicates the second policy intervention time point (March 2021)


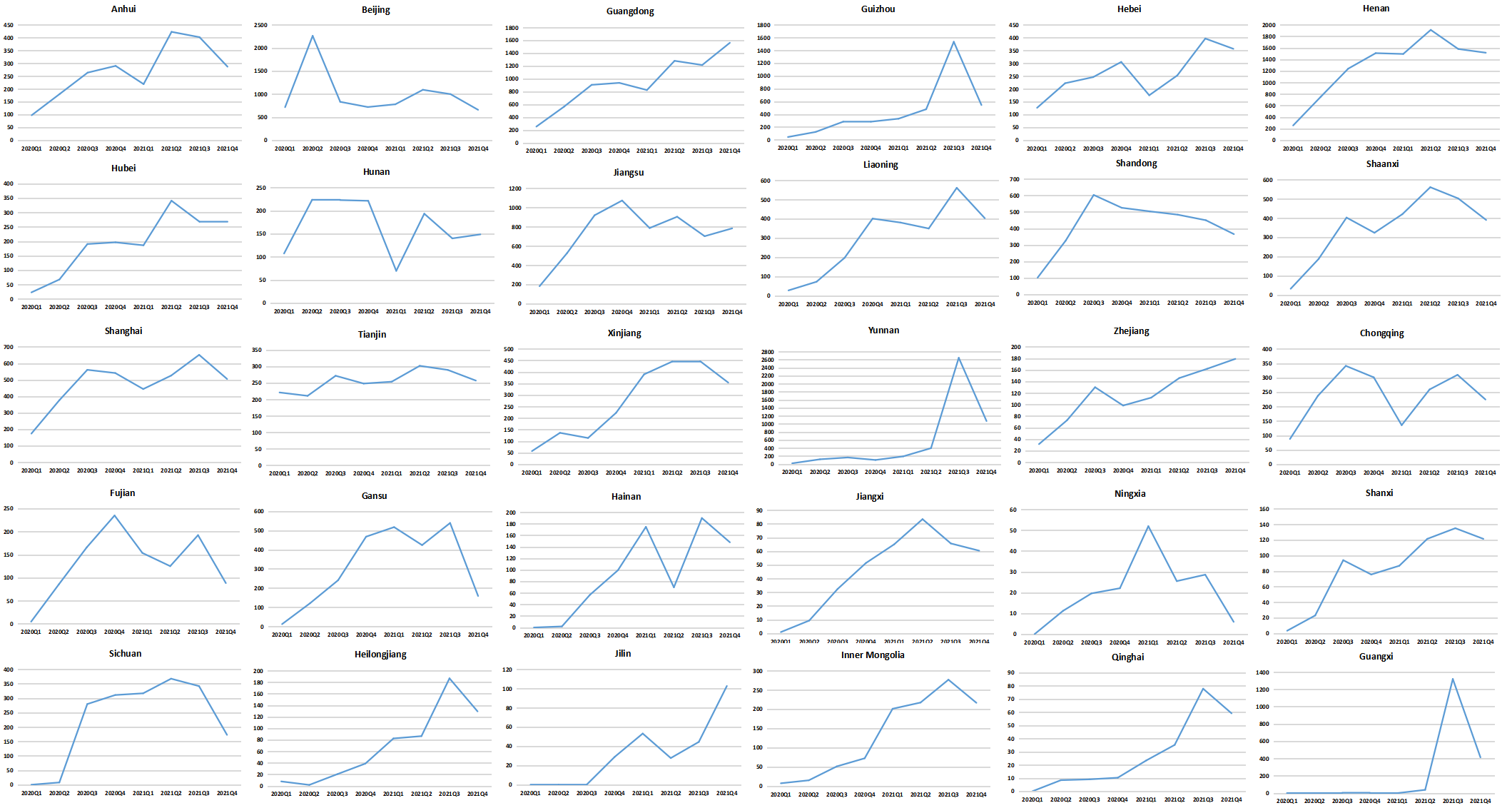


Annex 5 Quarterly number of 3-month standard DAA treatment of hepatitis C at provincial level (Q1 2020–Q4 2021)

Annex 6 Model parameter of Latent Class Trajectory Models

|  | classes | AIC^a^ | BIC^b^ | entropy |
| --- | --- | --- | --- | --- |
| N=30 | 1 | 198.11 | 212.12 | 1.00 |
|  | 2 | 184.02 | 203.64 | 1.00 |
|  | 3 | 181.61 | 206.23 | 0.96 |
|  | **4** | **179.70** | **210.52** | **0.93** |
|  | 5 | 183.43 | 219.86 | 0.92 |
|  | 6 | 186.93 | 228.97 | 0.87 |
|  | 7 | 185.42 | 233.06 | 0.89 |

**Note:** ^a^ AIC: Akaike Information Criterion；^b^ BIC: Bayesian Information Criterion
